# Searching for ivermectin resistance in a Strongylidae population of horses stabled in Poland

**DOI:** 10.1186/s12917-017-1133-1

**Published:** 2017-07-03

**Authors:** A. Zak, N. Siwinska, M. Slowikowska, H. Borowicz, K. Kubiak, J. Hildebrand, M. Popiolek, A. Niedzwiedz

**Affiliations:** 1 Department of Internal Medicine and Clinic of Diseases of Horses, Dogs and Cats, Faculty of Veterinary Medicine, Wroclaw University of Environmental and Life Sciences, pl. Grunwaldzki 47, 50-366 Wroclaw, Poland; 2 Department of Parasitology, Institute of Genetics and Microbiology, University of Wroclaw, ul. S. Przybyszewskiego 63/77, 51-148 Wroclaw, Poland

**Keywords:** Strongylidae, Horses, Ivermectin resistance, Ivermectin effectiveness

## Abstract

**Background:**

There are no available studies describing the possible resistance of strongyles to ivermectin in horses in Poland. One hundred seventy three horses from nine stud farms from South-Western Poland were studied. The effectiveness of ivermectin was studied on the 14th day after ivermectin administration using the fecal egg count reduction test, and a long-term observation of the egg reappearance period was carried out. The fecal study was carried out using a modified McMaster method, which typically detects 20 eggs per gram of stool. The results were subjected to statistical analysis that enabled quantification of the eggs in the stool samples.

**Results:**

The study revealed high efficacy of ivermectin on the 14th day after administration without a shortening of the egg reappearance period.

**Conclusion:**

The results indicate that strongyles resistance to ivermectin in Poland is not a serious problem.

## Highlights

- Our results indicate that the efficacy of ivermectin measured on the 14th day after treatment is very high in horses stabled in Poland.
- There was no shortening of the egg reappearance period after ivermectin administration.
- Our results indicate that there is no ivermectin resistance in the strongylidae population in horses bred in South-Western Poland

## Background

Strongyles are considered to be major gastrointestinal parasites in horses [1, 2]. Pasture grazed adult horses are usually infected with strongyles [1, 3, 4]. The clinical symptoms associated with a strongyle infection are often non-specific. They include a weight loss, poor quality horse hair, diarrhoea and recurring colic [1, 5]. The migration of the fourth-stage small strongyle larvae (L4) and their encystation in the intestinal wall may lead to “larval cyathostomosis”. This, in turn, causes protein-losing enteropathy and diarrhoea [1, 6]. Anthelmintic drugs from three groups - the benzimidazoles (e.g. fenbendazole and oxibendazole), the tetrahydropyrimidine pyrantel and the macrocyclic lactones (ivermectin and moxidectin) are used in horses to treat strongyle infections. Long-term use of anthelmintic drugs may trigger parasite resistance in a given population. In the case of small strongyles (cyathostomins), benzimidazole resistance was reported in 14 countries while pyrantel resistance occurred in 12 countries [7]. Unlike benzimidazole and pyrantel, the efficacy of macrocyclic lactones is reported to be 99% [8]. However, there have been isolated cases of ivermectin resistance based on the fecal egg count reduction test (FECRT) and/or a shortened egg reapperance period (ERP) what is the first indicator of strongyle resistance to ivermectin [4, 8–15]. To date, the ivermectin resistance of strongyles in Poland has not been studied. The aim of the study was the assessment of ivermectin resistance of strongylidae in horses bred in Poland, considering routine horse deworming twice a year using ivermectin, without coproscopy.

## Methods

### Study design

The study was carried out in the first half of 2016 in nine stables in Lesser Poland, Greater Poland and Lower Silesian Voivoideships. In total, 173 horses of both genders and various breeds, with a mean age of 11.5 years (range: 1.5–32) were studied. The first part of the study, which included an evaluation of the efficacy of ivermectin on the 14th day after its administration, using the FECRT, was carried out on all 173 horses. In the second part of the study, the egg reappearance period (ERP) was assessed in 42 horses, from two stables, selected based on an EPG value exceeding 20. All the horses were routinely dewormed twice a year in accordance with current standards using single-component pastes in spring and two-component pastes in autumn. In both groups, the horses were naturally infected with strongyles. The study included pasture grazing horses which were dewormed more than 8 weeks prior to the commencement of the study [16, 17]. The examination at T0 consisted of collecting fresh stool samples from the litter bedding, evaluating the number of eggs per gram of stool during the fecal examination and determining the contamination potential of the horses. Deworming was carried out using an equine single-component oral paste (Ecomectin, Eco Animal Health) containing 18.7 mg/g of ivermectin administered at a dosage of 200 μg/kg. The body weight of the horses was recorded using a “girth tape”. Deworming was carried out by a qualified veterinary surgeon. No dose errors were reported. Stool samples were collected 14 days after deworming from all the horses. In addition, stool samples were collected on a weekly basis from 42 horses selected for the ERP, until at least 10% of the amount of eggs recorded at T0 was reached. The fecal examination followed a modified McMaster centrifugation – enhanced method using a sugar-salt flotation solution with a specific gravity of 1.3 g/ml [18, 19]. The detection limit of the method was 20 EPG [18].

### Statistical analysis

At T0, the contamination potential of the horses older than 3 years was determined using the 2013 AAEP Parasite Control Guidelines. In each yard, the horses were classified into groups based on the varying degree of strongyle egg shedding: low contaminators – EPG <200, moderate contaminators – EPG range: 201–500 and high contaminators - EPG >500 [4]. The chi square independence test was used to compare contamination potentials in different age and sex groups. Statistical significance was set at *P* < 0.05.

The primary outcome measure in this study was the analysis of the length of the ERP. The ERP was defined as the time between the treatment and the first breaching of a 90% efficacy threshold [20].

The secondary outcome measure was the percentage reduction in the arithmetic mean of the strongyle FEC on day 14 (after) relative to day 0 (before): (FEC_D0_ – FEC_D14_)/FEC_D0_ with the 95% confidence interval (CI). Anthelmintic efficacy was defined as a reduction in FEC ≥95% with the lower 95% CI >90% was considered as efficacious [21, 22]. Anthelmintic resistance was present if (1) the percentage reduction in FEC was ≤95% and (2) the lower 95% CI was <90%. Suspected anthelmintic resistance was present if (1) the percentage reduction in FEC was ≤95% or (2) the lower 95% CI was ≤90%.

In addition, the results of the efficacy of ivermectin on the 14th day after its administration underwent statistical analysis using a specifically designed “eggCounts” package in R (using Bayesian hierarchical models for faecal egg count data) and a web interface, (http://www.math.uzh.ch/as/index.php?id=eggCounts) enabling the elimination of the Poisson errors in the counting technique. [23]. A correction factor = 20 and zero-inflation were used in the calculations.

## Results

All the animals were dosed correctly, and no abnormal health events linked to the treatment were recorded in this study. The mean EPG in the horses in this study was 131.56 (CI = 95.58–167.55) and ranged from 0 to 2300. The contamination potential of the horses older than 3 years was classified as 78.48% - low contaminators, 15.69% moderate contaminators and 5.83% high contaminators. There was no statistically significant correlation between the sex, the age and the contamination potential in the studied horses (*p* = 0.05). No *Parascaris equorum* eggs were detected in the feces.

**Fig. 1:**
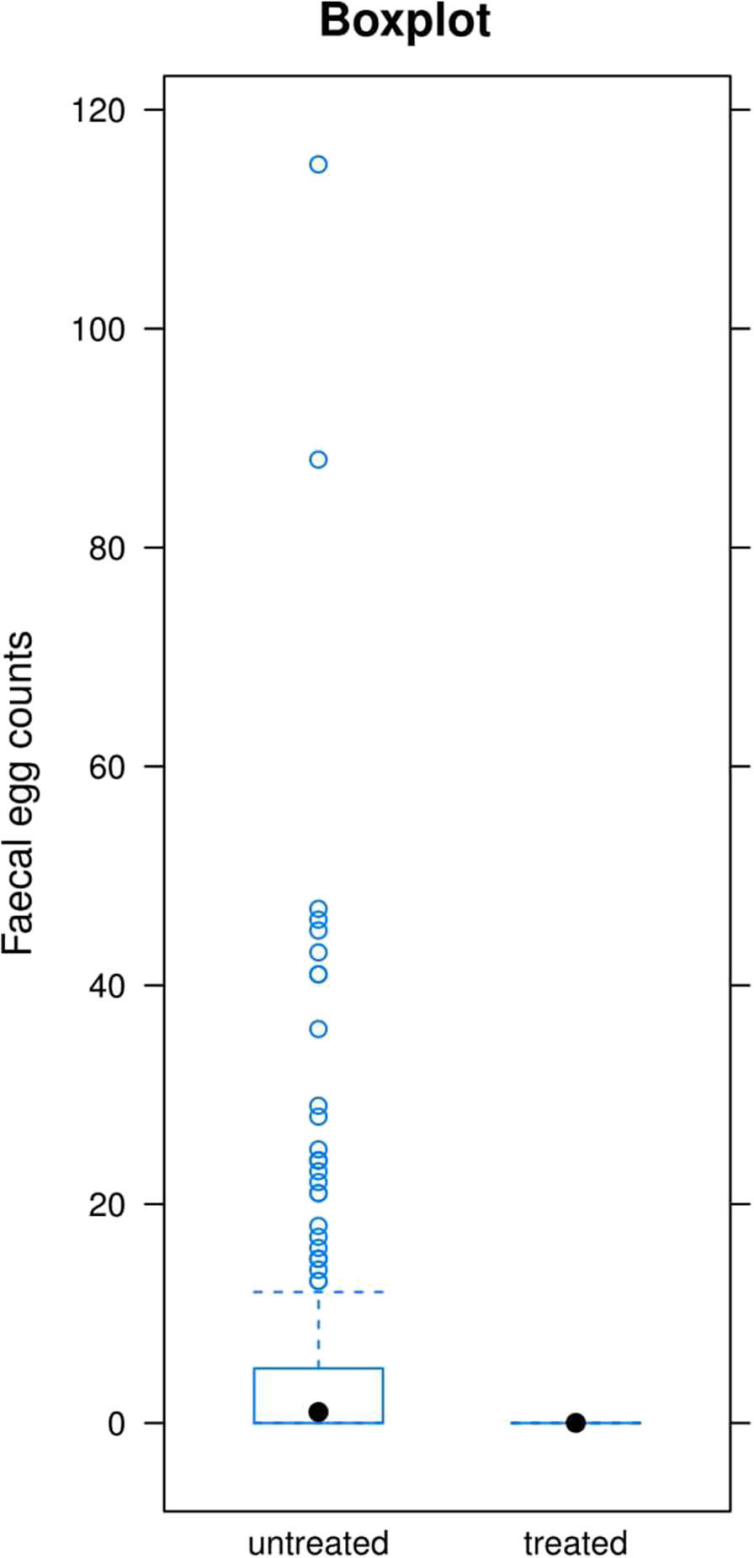
The graphical display contains information regarding the amount of EPG prior to and after treatment of all the horses

The analysis of the efficacy of ivermectin on the 14th day after its administration indicated that it was effective in 99.9% (CI = 95%). Detailed results are presented in Table 1. The results are presented in the form of graphs in Fig. 1.

**Table 1:** Summay statistics for each variable - generated using an “eggCounts” package in R (http://www.math.uzh.ch/as/index.php?id=eggCounts)

	2.5%	50%	97.5%
fecr	0.997	0.999	1
meanEPG.untreated	123.606	131.308	140.243
meanEPG.treated	0.003	0.078	0.433

The ERP ranged from seven to 16 weeks from the time of ivermectin administration. The ERP was not shortened, and the complete results are presented in Table 2.

**Table 2:** Results of ERP study – normal range for ivermectin is 42–56 days

Farm	number of horses	mean EPG	EPG range	ERP (days) – number of horses									
				D49	D56	D63	D70	D77	D84	D91	D98	D105	>D105
1.	14	350	20–1760	0	1	1	1	3	2	0	1	3	3
2.	28	92.14	20–900	1	5	8	3	6	1	1	1	0	2

## Discussion

The modified centrifugation – enhanced McMaster method according to Roepstoff and Nansen (1998) is considered the most accurate McMaster fecal egg counting technique in horses [19]. However, it is not possible to avoid errors associated with the choice of the fecal examination method. Hence, a specifically designed “eggCounts” package in R and a user friendly web interface were used to eliminate errors associated with the inaccuracy of the test method as well as the uneven and random distribution of the eggs in the stool sample [23]. That allowed an accurate interpretation of the results, which were more reliable. Our results indicate a high efficacy of ivermectin on the 14th day after its administration. Other reports confirm our findings and state an ivermectin efficacy of almost 100% on the 14th day after administration, or describe single cases of partial or total resistance [8–15]. The first indicator of strongyle resistance to ivermectin is a shortened ERP [4, 24]. It is difficult to compare the results of studies assessing the ERP, due to a lack of a uniform definition of the ERP. The EPG after treatment, which is considered a criterion determining the ERP, varies. Some investigators calculate the ERP as the first week in which eggs appear in the stool [25–28]. Other authors consider the EPG as 100 [16], 200 [29], <80% of the FECR [30] or <90% of the FECR [31–33]. In our study, ERP was considered to be the time a < 90% FECR was obtained. Unshortened ERP and a high efficacy of ivermectin on the 14th day after its administration rule out strongyle ivermectin resistance in horses bred in Poland. In order to slow down the development of resistance, it seems necessary to introduce a selective therapy in horses that have an egg per gram count exceeding 500, proposed by the 2013 AAEP Parasite Control Guidelines, or the deworming of horses that either have a FEC exceeding 200 EPG or that shed *Parascaris equorum* eggs*,* which is used in Denmark [20]. This study was prompted by reports of incorrect use of horse deworming programmes in Poland. Although this data is not available in literature, reports of incorrect deworming were received from several horse breeders and owners. The use of off label ivermectin in horses orally, intended for intravenous or intramuscular use in pigs and ruminants, may cause ivermectin-resistance. There are no reports of the pharmacokinetic and pharmacodynamic properties of intravenous or intramuscular ivermectin administered orally. Ivermectin was most probably used off label for economical reasons. Currently, great emphasis is placed on deworming procedures in Poland. Veterinary professionals and horse owners are encouraged to use selective deworming schemes in order to limit future ivermectin resistance.

## Conclusions

We found no strongyle ivermectin-resistance in horses bred in Poland based on the high efficacy of ivermectin on the 14th day after its administration and no shortening of the ERP. Despite these results, it is necessary to undertake measures that will limit future strongyle resistance, such as the use of correct deworming methods and selective deworming.

## Abbreviations

EPG: Egg Per Gram
ERP: Egg Reappearance Period
FEC: Fecal Egg Counts
FECR: Fecal Egg Count Reduction
FECRT: Fecal Egg Count Reduction Test
